# Obstetric fractures in caesarean delivery and risk factors as evaluated by paediatric surgeons

**DOI:** 10.1007/s00264-022-05547-2

**Published:** 2022-08-19

**Authors:** Alexandru Ulici, Alexandru Herdea, Mihai-Codrut Dragomirescu, Claudiu N. Lungu

**Affiliations:** 1 11th Department of Pediatric Orthopedics, “Carol Davila” University of Medicine and Pharmacy, Bd. Eroii sanitari nr. 8, 050474 Bucharest, Romania; 2 Pediatric Orthopedics Department, “Grigore Alexandrescu” Children’s Emergency Hospital, 011743 Bucharest, Romania; 3 Department of Surgery, Clinical Country Emergency Hospital Galati, 800008 Galati, Romania

**Keywords:** Obstetric fractures, Clavicle fractures, Cesarean delivery

## Abstract

**Introduction:**

Obstetric fractures usually occur after complicated births and are sent to paediatric orthopaedics for treatment and follow-up. Clavicle fractures represent the most common orthopaedic birth injury, involving approximately 0.2 to 3.5% of births.

**Hypotheses:**

Caesarean delivery, elective or necessary, along with the type of presentation, may play a determinant role in the aetiology of obstetric fractures.

**Materials and methods:**

We chose to do a retrospective study to determine possible risk factors for this type of injury that may manifest in either delivery. Our aim was to deepen our knowledge in order to have a better prediction and a better management of this condition. Data gathered included parity, gestity, type of delivery, presentation, shoulder dystocia, type of fracture, birth weight, and APGAR score.

**Results:**

We followed 136 patients that were diagnosed with Allman type I clavicle fracture, 32 of them also having brachial plexus birth palsy (BPBP) type 1 (Duchenne-Erb). Natural birth with a pelvic presentation imposes a relative risk of 6.2 of associated pathology compared to cranial presentation. Caesarean delivery and cranial presentation increase the risk of related pathology by 5.04 compared to natural birth. Statistically, pelvic presentation is 5.54 times more likely to develop related pathology than cranial presentation. Type of delivery and presentation correlate with associated pathology of clavicle fractures.

**Discussion and conclusion:**

Caesarean delivery brings risks for the newborn and should be practiced only when necessary. Predictive modeling in obstetrics in third-trimester evaluations may statistically predict risks of birth complications like fracture and BPBP.

## Introduction

Obstetric fractures are fractures that newborns suffer during the delivery or birth process. The most commonly affected bones are the clavicle, the humerus, the femur, and the skull [1, 2]. Clavicle fractures represent the most common orthopaedic birth injury, involving approximately 0.2 to 3.5% of births, with equivalent sex distribution and laterality [3, 4]. The pathophysiology of obstetric fractures remains uncertain^3^. Although they occur more often during vaginal delivery, most probably due to compression of the anterior fetal shoulder against maternal symphysis pubis after delivery of the head (shoulder dystocia), they can also occur in cesarean deliveries [5]. The most frequent risk factors incriminated in prior studies are macrosomia, instrumented delivery, post-term delivery, shoulder dystocia, length more than 52 cm, APGAR score, prolonged labor [6], and the skills of the obstetrician [7]. On the mode of delivery, one study suggested that clavicle fractures occur most commonly in vaginal delivery, whereas fractures of the long bones tend to complicate caesarean section [8]. Related to the mode of presentation, the cephalic is associated with clavicle and skull fractures, and the breach is related to femoral and humeral fractures[9].

Allman classifies fractures of the clavicle in three groups [10]. Group I fractures are the most common type and involve the middle third of the clavicle. Fractures of the distal third belong to group II. Allman group III, the rarest kind of all three, comprises fractures of the distal third of the clavicle. Clinical findings include tenderness, decreased Moro reflex, crepitation, swelling of the affected shoulder, and an X-ray exam confirming the diagnostic [11, 12].

Clavicular fractures usually heal without complications or late sequelae [13]. The literature shows an association between clavicle fractures and obstetric brachial plexus palsy in 4–13% of cases, 90% of these cases consist of a transitory deficit. Other studies suggest that brachial plexus birth palsy and clavicle fracture occur concurrently and in isolation [14]

The decisive role of the type of birth and the presentation in the etiology of neonatal clavicle fractures is well known. Our study aims to determine possible risk factors for this type of injury that may manifest in either delivery to predict better and manage this condition.

## Materials and methods

We set out a retrospective study regarding children who presented to the Paediatric Orthopaedic ward of “Grigore Alexandrescu” Children’s Emergency Clinical Hospital of Bucharest from 2018 to 2020 with obstetric fractures. The ethics committee of “Grigore Alexandrescu” Children’s Emergency Clinical Hospital of Bucharest approved this study on 14 December 2021. The identification number of the survey is 34/14 December 2021. Informed consent was obtained from the parents of all the participants when they presented initially.

The main keywords we searched in our database were “obstetrical fracture” and “brachial plexus lesion” on infants under one year of age. The inclusion criteria were positive diagnosis of an obstetrical fracture with clinical and radiological findings, neurological examination of the affected limb, parental consent, and complete patient history. Exclusion criteria were non-obstetrical fractures (accidental fractures, traffic collision trauma, Silverman’s syndrome, or child abuse syndrome), lack of data regarding delivery and labour, lack of radiological examination that might confirm the diagnostic, and lack of parental consent.

**Fig. 1:**
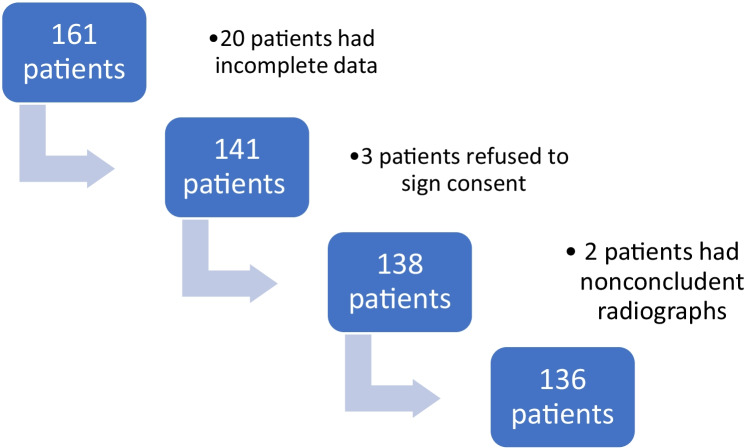
Flow diagram of the patients included in the study. After inclusion and exclusion criteria were applied, 136 patients remained in the study

From the initial group of 161 patients, 20 patients lacked a complete patient history. Three patients did not have the parental consent to be included in the study, and two patients had inconclusive radiological exams. Only 136 patients met the inclusion criteria (Fig. 1).

The following variables were analyzed in search for statistical significance: maternal age, height, antepartum weight, weight gain after pregnancy, urban/rural environment, parity, gestity, place of birth, public/private hospital, type of delivery, gestational age, presentation, shoulder dystocia, type of fracture, birth weight, gender, duration of labour and expulsion, APGAR score, and complicated/straightforward fracture.

A *p*-value less than 0.05 was considered statistically significant, with a corresponding confidence level of 95%. Statistical analysis was carried out using Microsoft Excel 2016 and RStudio.

## Results

Out of 136 patients, 68 were boys and 68 were girls. All of them were diagnosed with Allman I clavicle fracture; 32 children (14 girls, 18 boys) were also diagnosed with brachial plexus birth palsy (BPBP) type 1 (Duchenne-Erb) involving C5 and C6 nerves. Restitutio ad integrum was the outcome of every patient with BPBP until 2. Our study affirms that gender was not a risk factor for obstetrical fractures (Pearson’s chi-squared test—*p* = 0.4187). Birth weight did not correlate with gender statistically significantly, with a mean girls’ weight of 3418 g and a mean boys’ weight of 3510 g (*T*-test, *p*-value = 0.21).

Place of birth and national/private healthcare were also noted but did not correlate with fracture incidence.

Regarding the type of delivery, 99 cases were vaginal births and 36 were cesarean deliveries. Out of them, 25 were elective cesarean deliveries, and 11 were necessary due to fetal macrosomia (4), preeclampsia and maternal hypertension (1), maternal thrombocytopenia (4), and postmaturity (3) (Table 1).

**Table 1:** Patient distribution regarding the type of delivery and comorbidities (BPBP)

	Type of delivery		
Presence of comorbidities	Cesarean	Natural	Total
Yes	17	15	32
No	20	84	104
Total	37	99	136

In concordance with the independence test, a *p*-value of 0.0001648 shows enough proof that the type of delivery correlates with associated pathology of clavicle fractures.

We built a logistic regression model to assess the causality relationship between these two variables. According to the logistic regression, the type of delivery correlates with fracture complications statistically (*p*-value = 0.000313). Thus, cesarean delivery increases the risk of birth-related complications 4.75-fold compared to natural birth. This result only considers the type of birth and no other possible risk factors.

There were 124 children with the cranial presentation, from which 99 had no associated pathologies and 25 developed related pathology. There were 12 children with the pelvic display, and seven had associated pathology. After performing logistic regression, we found that presentation correlates with associated pathology statistically (*p*-value = 0.00629). We discovered that pelvic presentation is 5.54 times more likely to develop related pathology than cranial presentation.

Because the type of presentation and the type of delivery had proven to be relevant in the development of the associated pathology, we conducted a model of logistic regression that includes them both, and we found that regarding cranial presentation, caesarean delivery increases the risk of related pathology by a factor of 5.04 compared to natural birth. Also, about natural birth, a pelvic presentation imposes a relative risk of 6.2 of associated pathology compared to cranial presentation.

Next, we assessed maternal age and birth weight in correlation with the risk of fractures. We classified the neonates into four groups according to their birth weight: small (< 3000 g), average (3000–3800 g), large (3800–4200 g), and huge (> 4200 g). In our study, maternal age did not represent a statistically significant variable (*p*-value = 0.72) instead of birth weight.

Maternal height and antepartum weight, as well as postpartum weight, have been expressed as BMI (body mass index). According to our analysis, the risk of associated pathology increases by 6% for each increment in maternal BMI during pregnancy for a particular child’s weight. We also found that for a certain maternal BMI, the risk of associated pathology decreases by 83% for each kilogram over the minimum birth weight in our group of children. Increased birth weight has proven to be a protective factor against the associated pathology.

After conducting a logistical regression with birth weight and type of delivery as variables, we found that they correlate statistically. Caesarean delivery imposes a relative risk of 9.6 of associated pathology compared to vaginal delivery for a certain birth weight.

The model based on birth weight and type of delivery varies according to the confidence intervals that we choose. For example, for a newborn with a weight of 3.46 kg, delivered vaginally, there is a probability of 9% of associated pathology. In contrast, for a newborn with the same weight provided through the caesarean section, the chance is 51.9%.

**Fig. 2:**
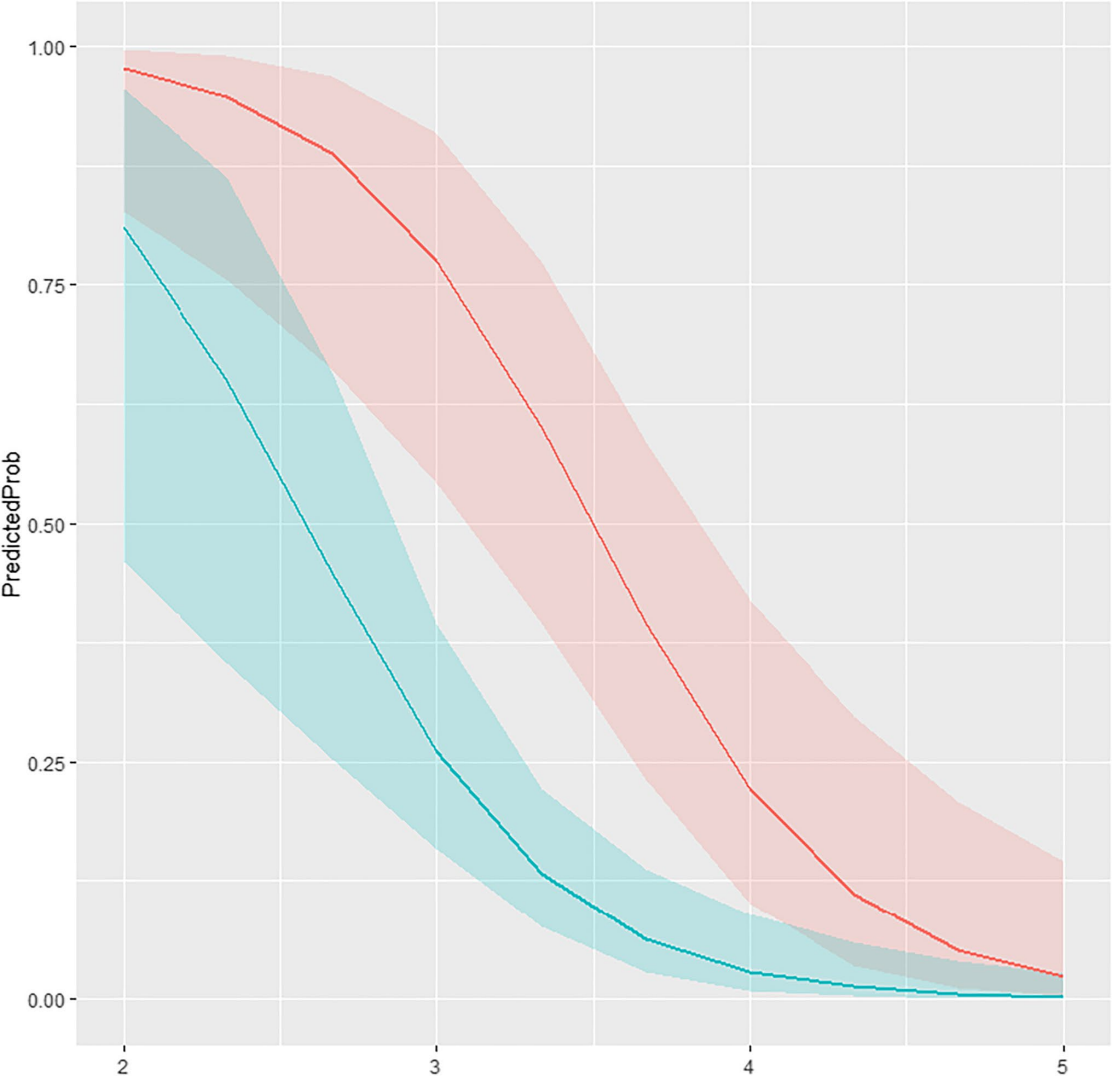
Graphic illustration of predicted probabilities (red flow—cesarean, green flow—natural birth)

The following table and figures (Table 2 and Fig. 2) show the estimative probability of associated pathology depending on birth weight and type of delivery, including the upper and lower limits of confidence intervals.

**Table 2:** Estimation and prediction probability for an associated pathology to happen, depending on birth weight and type of delivery

Nr	Weight	Type of delivery	Predicted prob	Lower	Upper
*1*	0.810420333	Natural	0.810420333	0.4604767931	0.95537931
*2*	2.333333	Natural	0.650435065	0.3541931470	0.86325147
*3*	2.666667	Natural	0.447484537	0.2535260786	0.65885932
*4*	3.000000	Natural	0.260642658	0.1593840601	0.39593209
*5*	3.333333	Natural	0.133030822	0.0764747729	0.22138594
*6*	3.666667	Natural	0.062607667	0.0274371154	0.13653289
*7*	4.000000	Natural	0.028249983	0.0085685742	0.08907653
*8*	4.333333	Natural	0.012495687	0.0025429705	0.05909375
*9*	4.666667	Natural	0.005477637	0.0007396086	0.03937216
*10*	5.000000	Natural	0.002391642	0.0002131340	0.02625266
*11*	2.000000	Cesarean	0.976596535	0.8270509509	0.99726125
*12*	2.333333	Cesarean	0.947816667	0.7553157155	0.99072973
*13*	2.666667	Cesarean	0.887714592	0.6613582301	0.96970061
*14*	3.000000	Cesarean	0.774834680	0.5424078916	0.90900860
*15*	3.333333	Cesarean	0.599653899	0.3959259413	0.77390889
*16*	3.666667	Cesarean	0.394659356	0.2314273826	0.58533958
*17*	4.000000	Cesarean	0.221049660	0.1001123947	0.41990985
*18*	4.333333	Cesarean	0.109940257	0.0347022795	0.29795150
*19*	4.666667	Cesarean	0.051021305	0.0108168688	0.20907444
*20*	5.000000	Cesarean	0.022866851	0.0032243031	0.14479029

Following a logistic regression between parity as a variable and the presence of a fracture/associated pathology, we found that multiparae have a 90% lower risk of newborn injuries than primiparae.

Following the logistic regression with associated pathology as a variable and also birth weight, parity, and type of delivery, we found that all three quotients are statistically significant (mentioning that in this case, equality has a limited statistical significance with a *p*-value of 0.0089).

This logistic regression model shows that multiparity decreases the risk of birth injuries by 84% compared to primiparity for a particular birth weight and a specific type of delivery. Each additional kilogram of birth weight reduces the risk of fractures/associated pathology by 91% for a certain parity and a particular kind of delivery. Also, cesarean delivery has a chance of 7.82 times higher than natural birth for a certain weight and equality.

## Discussion

A clavicle fracture is a relatively benign, common birth injury. The main concern of this condition is the presence/absence of complications. Hence, it is still a valuable object of study in both orthopaedics and obstetrics. Most scientific literature agrees about the known risk factors of neonatal clavicle fractures: macrosomia, primiparity, type of delivery, labour time, APGAR score, presentation, maternal age, and BMI. Our results are mostly consistent with others’, nuanced by a thorough statistical analysis.

Their ten year retrospective study about clavicle fractures among vaginally delivered babies reported that the birthweight of the infants that suffered clavicle fractures was significantly higher (*p* < 0.001), and the head to chest circumference ratio was considerably lower. They also note that APGAR scores at 1 and 5 min are significantly lower in the fracture group [15]. Choi et al., in their 12-year period retrospective review of caesarean deliveries with a neonatal clavicle fracture, note that macrosomia is also a risk factor for this type of trauma among babies born through abdominal delivery [16]

Unlike other studies, we found increased birthweight over a recorded minimum to be a protective factor against birth-related trauma and its complications. Our result came after analyzing each newborn weight rather than establishing a threshold, and this could be a complementary finding to literature rather than a source of debate. As per macrosomia, we only counted four cases among the study group, and we had no control group to compare with [17, 18].

Their 12-year retrospective study of vaginal deliveries of liveborn infants in vertex presentation concluded that low parity, forceps delivery, and a high birth weight increase the risk of delivering a neonate with a clavicle fracture. Turnpenny et al., who conducted a study in a population with an increased incidence of grand-multiparae, conclude that parity does not significantly affect incidence in the younger age group (mothers under 25). Likewise, age does not have a considerable impact on primigravid women. Therefore, the main effect is that parity and increasing age jointly result in a higher fracture incidence. Thus, fractures are more likely to occur if the mother is older and parous. Our study suggests that nulliparity is a risk factor for obstetric clavicular fractures compared to their results. According to our statistic analysis, multiparae have a 90% lower risk of newborn trauma than primiparae. In our study, maternal age did not represent a statistically significant variable in infant injury [19].

A study suggests that breech births are three times more common in the fracture group compared with the whole population. This type of presentation is a risk factor in our present study and already published literature. Furthermore, we found that pelvic presentation is 5.54 times more likely to develop pathology associated with the clavicle fracture than cranial presentation. Orthopaedic examination of breech newborns should obligatory include hip ultrasound, knowing that this condition is associated with developmental dysplasia of the hip [20].

Kalk et al. on the impact of maternal BMI on neonatal outcome note that overweight and obese mothers exhibit an increased risk of delivering via caesarean section compared to healthy weight mothers. In contrast, for underweight mothers, no such association is detected. Caesarean section can also be technically more difficult in obese women, and there is a higher risk of anaesthetic and postpartum complications than in normal-weight mothers. Also, overweight or obese mothers have a significantly increased risk of macrosomic (birth weight > 4000 g) children (OR about 1.5 and 2), whereas underweight mothers are not at risk. Furthermore, elevated maternal BMI is associated with increased admission to neonatal care and with a lower APGAR score at one and five minutes, according to Mansart et al. Our results support maternal BMI as an independent risk factor for newborn fractures and associated complications, raising the risk with 6% per each BMI increment [21].

The strength of this manuscript is a thorough statistical analysis, both univariate and multivariate. The study limitations were our sample size, which may undergo “The law of small numbers,” our complete lack of data about the obstetricians’ extraction manoeuvres, and their expertise (which we could not assess as a potential risk factor compared to other studies conducted in OB-GYN wards) [22].

In summary, the results of our study agree with other reports concerning obstetric trauma [23, 24]. For future thoroughgoing studies, we aim to collect a more extensive patient pool to increase the statistical significance of our analyses and bring novelty to this field of research by finding new risk factors.

## Conclusion

Caesarean delivery brings risks for the newborn and should be practiced only when necessary. It is possible to use predictive modeling in obstetrics in third-trimester evaluations. One may statistically predict risks of birth complications like fracture and BPBP. More data is needed, from larger study samples, to find new risk factors and validate our logistic regression models.
